# Live and Recorded Music Interventions to Reduce Postoperative Pain: Protocol for a Nonrandomized Controlled Trial

**DOI:** 10.2196/40034

**Published:** 2023-03-10

**Authors:** Eleanor E Harding, Hanneke van der Wal-Huisman, Barbara L van Leeuwen

**Affiliations:** 1 Department of Surgery University Medical Center Groningen University of Groningen Groningen Netherlands; 2 Department of Otorhinolaryngology University Medical Center Groningen University of Groningen Groningen Netherlands; 3 Prince Claus Conservatory Hanze University of Applied Sciences Groningen Netherlands

**Keywords:** live music intervention, recorded music intervention, pain VAS, postsurgical pain, neuroinflammation, pain management, intervention, music, postoperative, pain, perception, pain relief, live music, recorded music, music intervention, music, pain perception

## Abstract

**Background:**

Postoperative patients who were previously engaged in the live musical intervention Meaningful Music in Healthcare reported significantly reduced perception of pain than patients without the intervention. This encouraging finding indicates a potential for postsurgical musical interventions to have a place in standard care as therapeutic pain relief. However, live music is logistically complex in hospital settings, and previous studies have reported the more cost-effective recorded music to serve as a similar pain-reducing function in postsurgical patients. Moreover, little is known about the potential underlying physiological mechanisms that may be responsible for the reduced pain perceived by patients after the live music intervention.

**Objective:**

The primary objective is to see whether a live music intervention can significantly lower perceived postoperative pain compared to a recorded music intervention and do-nothing control. The secondary objective is to explore the neuroinflammatory underpinnings of postoperative pain and the potential role of a music intervention in mitigating neuroinflammation.

**Methods:**

This intervention study will compare subjective postsurgical pain ratings among 3 groups: live music intervention, recorded music intervention, and standard care control. The design will take the form of an on-off nonrandomized controlled trial. Adult patients undergoing elective surgery will be invited to participate. The intervention is a daily music session of up to 30 minutes for a maximum of 5 days. The live music intervention group is visited by professional musicians once a day for 15 minutes and will be asked to interact. The recorded music active control intervention group receives 15 minutes of preselected music over headphones. The do-nothing group receives typical postsurgical care that does not include music.

**Results:**

At study completion, we will have an empirical indication of whether live music or recorded music has a significant impact on postoperative perceived pain. We hypothesize that the live music intervention will have more impact than recorded music but that both will reduce the perceived pain more than care-as-usual. We will moreover have the preliminary evidence of the physiological underpinnings responsible for reducing the perceived pain during a music intervention, from which hypotheses for future research may be derived.

**Conclusions:**

Live music can provide relief from pain experienced by patients recovering from surgery; however, it is not known to what degree live music improves the patients’ pain experience than the logistically simpler alternative of recorded music. Upon completion, this study will be able to statistically compare live versus recorded music. This study will moreover be able to provide insight into the neurophysiological mechanisms involved in reduced pain perception as a result of postoperative music listening.

**Trial Registration:**

The Netherlands Central Commission on Human Research NL76900.042.21; https://www.toetsingonline.nl/to/ccmo_search.nsf/fABRpop?readform&unids=F2CA4A88E6040A45C1258791001AEA44

**International Registered Report Identifier (IRRID):**

PRR1-10.2196/40034

## Introduction

In recent research, postoperative patients exposed to a live musical intervention reported significantly reduced perception of pain than patients without the intervention [1]. This encouraging finding indicates a potential for postsurgical musical interventions to have a place in standard care as therapeutic pain relief. However, live music is logistically complex in hospital settings, and previous studies have reported the more cost-effective *recorded* music to serve as a similar pain-reducing function in postsurgical patients [2,3]. Therefore, this study primarily aims to compare the effectiveness of postoperative live versus recorded music interventions in reducing patients’ perception of postsurgical pain. In order to potentially expand this finding from subjective to objective metrics, secondary outcomes will explore whether subjective pain perception is statistically related to more objective metrics such as pain medication dosage or respiration rate [4].

Although previous findings showed a convincing influence of music intervention on postsurgical pain perception [1-3,5-7], the physiological mechanisms that could make this possible are not yet understood [8]. It has been reported that music modulates psychoneuroimmunology [9,10] such as lowering stress-induced cortisol [11] and enhancing neurogenesis [12]. While chronic or long-term postsurgical pain has established links to neuroinflammation [13,14], the possibility exists that immediate postsurgical pain—before it reaches a chronic state—may have links to neuroinflammation as well [15]. Thus, one possibility is that music is mitigating postsurgical neuroinflammation, which could in turn be affecting the immediate postsurgical pain.

Therefore, further secondary outcomes will explore behavioral indicators (postoperative cognitive decline or POCD and delirium) and biological markers (interleukin-6 [IL6], C-reactive protein [CRP], neutrophil gelatinase–associated lipocalin [NGAL], and translocator protein [TPSO]) with established neuroinflammatory underpinnings [16-20] to see whether the primary pain outcome has a statistical relationship to these neuroinflammatory-related metrics. The exploratory electroencephalogram (EEG) analyses will also be conducted to see whether either entropy [21], alpha power [22], or delta/alpha power ratio [23,24] can provide neuroinflammatory markers, which could correlate with postoperative pain outcomes. Finally, we will explore whether these neuroinflammatory markers seem to be mitigated by either the live music or active control intervention to explain the reduced pain reported by postsurgical patients in music intervention studies [2,20].

The primary objective is to see whether a live music intervention reduces pain perception compared to both recorded music active control and standard care control groups. The secondary objective is to explore the role of music in mitigating the neuroinflammatory response to surgery, for future hypothesis-directed research.

## Methods

### Study Design

The study will take place at the University Medical Center Groningen (UMCG), which has already hosted the Meaningful Music in Healthcare (MiMiC) live intervention [25]. The intervention study follows an on-off nonrandomized controlled trial (NCT) design [26]. The NCT is necessary for this particular intervention in practice because the live music intervention cannot easily be randomized to skip patients in the same ward without them hearing the music or seeing the musicians as they play for other patients and nurses.

In this NCT design, treatments and data collection will be conducted in 15 blocks over the course of 4 years. According to the 29-day treatment block, the three 5-day treatments (live music intervention, recorded music active control intervention, and standard care control) will be offered with a 7-day washout between treatments. The washout will minimize the number of participants whose hospital stay overlaps with another treatment. Based on the previous research (UMCG research register 201600541), we expect approximately 8 full data sets per 5-day treatment block.

Note that while the intended outcome of the music intervention is therapeutic (eg, experiencing reduced pain), the music intervention is conducted by conservatory-trained music practitioners but not by music therapists. Music therapists are licensed therapists who have a degree in music therapy including clinical training. The music practitioners in this study are primarily professional musicians who have taken 1 music-conservatory course and several internships to train them for clinical settings but they are not therapists.

**Figure 1:**
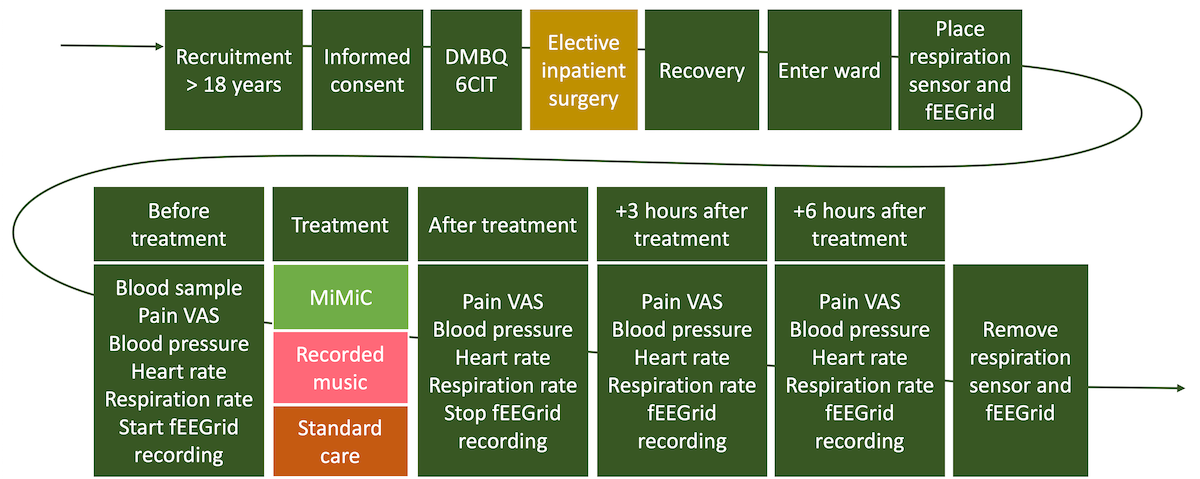
Chronological walk-through of the design procedures. After entering the ward, subsequent steps repeat per each day of treatment. Blood sample, blood pressure, and heart rate are part of standard care measurements and will be incorporated with standard care whenever possible. 6CIT: 6-item cognitive assessment; DMBQ: Dutch Musical Background Questionnaire; fEEGrid: face electrode grid for electroencephalogram recordings; MiMiC: Meaningful Music in Healthcare; VAS: visual analog scale.

Design procedures are chronologically displayed in Figure 1. Preoperative questionnaires will be collected to assess cognitive function (6-item cognitive assessment [6CIT]) [27,28] and musical background (excerpt from the Dutch Musical Background Questionnaire [DMBQ]) [29,30]. Postoperative data collection procedures per day of treatment include pain visual analog scale (VAS) ratings, heart rate (HR), and blood pressure measuring 4 times a day (before, directly after, 3-hour, and 6-hour postintervention). EEG electrodes will be applied once in the morning and 4 EEG recordings will be collected with VAS ratings, HR, and blood pressure (before, during, 3-hour, and 6-hour postintervention). A blood sample will be collected once daily and integrated with standard care as far as possible (eg, if blood is drawn as part of standard care, the sample will be collected from the same needle). A schedule of assessments may be found in Table 1. Daily dosage of pain medication as well as any incidence of delirium (delirium observation screening scale [DOSS]) [31] or POCD will be collected.

**Table 1:** Schedule of procedures.

Time		Procedures	Duration (minutes)
Preoperative		Six-item cognitive assessment Dutch Musical Background Questionnaire excerpt	7.5 and 4.5 (total 12)
**Postoperative**			
	9 AM	fEEGrid^a^ preparation and placement Respiration biosensor placement Blood vial^b^	10
	10:30 AM	VAS^c^ rating, HR^d^, respiration rate, blood pressure, and start EEG^e^ recording	5
	10:40-11 AM	MiMiC^f^ or recorded music or no intervention VAS rating, HR, respiration rate, blood pressure, and stop EEG recording	20
	2 PM	VAS rating, HR, respiration rate, blood pressure, and EEG recording	15
	5 PM	VAS rating, HR, respiration rate, blood pressure, and EEG recording	15
	5:15 PM	Remove fEEGrid and respiration biosensor	5
	Total	N/A^g^	70

^a^fEEGrid: face electrode grid for EEG recordings.

^b^The blood vial collection will be integrated into standard care as much as possible, thus, the time may not be 9 AM if other blood collection is at a different time.

^c^VAS: visual analog scale.

^d^HR: heart rate.

^e^EEG: electroencephalogram.

^f^MiMiC: Meaningful Music in Healthcare.

^g^N/A: not applicable.

### Population (Base)

The study will include patients who have undergone elective surgery in the UMCG and are hospitalized for at least 1 day following surgery. All patients must be 18 years or older and able to make their own decisions. Patients must further be able to hear music with normal or corrected normal hearing (ie, a hearing aid).

### Inclusion Criteria

In order to be eligible to participate in this study, a subject must meet all of the following criteria: (1) 18 years or older, (2) postsurgery hospitalization (all surgery types), (3) elective surgery, (4) able to hear music, (5) able to give informed consent, and (6) able to communicate.

### Recruitment

Patients will be informed as soon as the date of hospital admission is set in the weeks that the study is planned. The patient will receive an information letter and informed consent from the provider or it will be sent to them by email. A member of the research team will contact the patients after a minimum of 24 hours for participation and answer possible questions. Patients will have up to the time of their surgery to decide, allowing for time to fill out the preoperative questionnaires. Consent can be withdrawn at any time at which point all data collection and treatment will stop.

### Ethics Approval

This protocol has been approved by the Central Medical Ethics Committee of the UMCG (METc2021.359) and is registered with the Netherlands central commission of human research (CCMO-register number NL76900.042.21). The informed consent letter is written in plain, nonacademic Dutch with no medical jargon. Only pseudoanonymized (deidentified) data will be published. Patients participating in the study will receive a small token gift (such as a hand lotion or lip balm).

### Study Parameters and End Points

#### Main Study Parameter and End Point

Subjective pain ratings are determined with a VAS rating (0-10) [1].

#### Secondary Study Parameters and End Points

The secondary study parameters (Table 2) and end points are as follows: (1) objective pain metric (volume of administered pain medication); (2) respiratory rate and tidal volume; (3) HR; (4) blood pressure; (5) neuroinflammation blood markers (IL6, NGAL, CRP, and serum TPSO); (6) behavioral indicators of neuroinflammation (delirium [31] and POCD incidence); and (7) EEG markers of neuroinflammation (alpha power, delta/alpha power ratio, and entropy).

**Table 2:** Secondary study parameters.

Outcome	Metric	Statistics
Objective pain measure	Pain medication dosage (ccs)	Chi-square test
Neuroinflammation, blood markers	IL6^a^, NGAL^b^, CRP^c^, and serum TPSO^d^	*F* test (RM^e^ ANOVA, pre-post)
Neuroinflammation, behavioral indication	Incidence delirium and incidence POCD^f^	*F* test (one-way ANOVA)
Neuroinflammation, EEG^g^ indication	Alpha power, alpha/delta power, and entropy	*F* test (RM ANOVA same as primary)
Neuroinflammation, physiological	HR^h^, respiration rate, and blood pressure	*F* test (RM ANOVA same as primary)

^a^IL6: interleukin-6.

^b^NGAL: neutrophil gelatinase–associated lipocalin.

^c^CRP: C-reactive protein.

^d^TPSO: translocator protein.

^e^RM: repeated measures.

^f^POCD: postoperative cognitive decline.

^g^EEG: electroencephalogram.

^h^HR: heart rate.

#### Other Study Parameters

Other study parameters used in this study are as follows: (1) comorbidities (Charlson comorbidity index) [32]; (2) complications (Clavien Dindo [33] and nurse-sensitive outcomes [34]); (3) postoperative days (T0=surgery); (4) surgery characteristics (invasiveness, etc) [20]; (5) musical background (excerpt from DMBQ) [30]; (6) age; and (7) presurgery cognitive testing [22] (eg, 6-item cognitive impairment score) [28].

### Treatment Allocation

This study will take the form of an on-off NCT [26]. An on-off NCT takes into account real-world setting limitations of certain intervention types and allocates participant inclusion by alternation. The main reason for this is that MiMiC is a well-researched intervention, whose practical implementation is not suited for randomly selected participants to not participate. On the one hand, musicians will visit the nurses’ station and play music in various rooms around the ward; thus, it is not possible to prevent participants who may be allocated non-MiMiC treatment from hearing the live music. On the other hand, a central tenet of the MiMiC intervention is inclusion and participation, thus “rejecting” patients who would want to participate in music they hear on the ward, on the basis of trial group allocation, goes against the spirit of the intervention.

In contrast, the NCT relies on the randomness of who is scheduled for surgery during a given intervention week but does not exclude any patient from the activity being conducted (listening to live or recorded music). Therefore, the treatment schedule (live music intervention, recorded music active control, and standard care control) will be predetermined months in advance, and the patients included will be offered whichever treatment is occurring during their hospital stay. The patients will, however, be asked for their consent before they are informed which treatment will be offered during their surgery recovery. This will attempt to prevent patients who are initially biased for inclusion based on their affinity to music or not.

### Study Procedures

After patients are recruited to the study, they will be allotted to the treatment occurring during the scheduled postoperative hospital stay. Noninvasive procedures include preoperative cognitive testing (6CIT), postoperative VAS pain ratings, HR, blood pressure, respiration rate, and EEG recording. Invasive procedures include obtaining blood samples, after which the serum will be analyzed in a laboratory for neuroinflammatory markers IL6, NGAL, CRP, and serum TPSO. An example schedule of procedures can be found in Table 1.

### Primary End Point

The main end point, VAS pain ratings, requires patients to subjectively rate their pain on a plastic VAS where they move a tick mark to the desired location on a 0-10 scale [1].

### Secondary End Points

The secondary end points, HR and blood pressure, will be collected from standard equipment on the ward at the time of obtaining VAS pain ratings.

#### Pulmonary Ventilation

Respiration rate and tidal volume have been linked to subjective pain experience in the literature [4]. Therefore, in order to see whether our pain findings extend from subjective to objective physiological measures, we will explore the association between these 2 objective metrics and subjective pain VAS ratings. A wearable biosensor that measures pulmonary ventilation metrics [35] will be placed on the chest of patients with an adhesive sticker and removed at the end of the day.

#### Blood Marker Analysis

One 10-ml vial of blood will be collected per day resulting in a maximum of 5 vials of blood over 5 days. The first sample must be taken before the first assigned intervention. Blood samples will be collected together with standard care insofar as possible. Serum extracted from blood samples will be processed by the Haemoscan lab and evaluated for the presence of 4 markers associated with neuroinflammation (IL6, NGAL, CRP, and serum TPSO; see “Introduction” section).

#### EEG Recordings

As opposed to typical electrode setup with, for example, a cap, the face electrode grids for EEG recording require minimal preparation of skin and are quickly applied. Each morning, the patient’s face will be cleaned and prepared with exfoliating gel, and electrodes will be applied with adhesive tape. The face electrode grid for EEG recordings will remain on the patient from morning until evening, spanning the first and last pain VAS ratings. Actual recordings will occur 4 times a day, in 15-minute increments, coinciding roughly with the times that pain VAS ratings are collected (before intervention, during intervention, 3-hour, and 6-hour postintervention). During the 15-minute EEG recording windows, the patients will be instructed to remain seated or lying doing a consistent activity of their choice, or otherwise as instructed during the assigned treatment.

#### Neuroinflammatory-Related Complications

After 30 days postsurgery, patient medical dossiers will be consulted, and any incidence of delirium and POCD will be recorded.

### Other Parameters

A short, 6-item questionnaire [27,28] will be added to the standard preoperative intake in order to assess preoperative cognitive status, as well as a short questionnaire about the musical background (excerpt from the DMBQ) [29,30]. Both the cognitive status and the musical background will help to interpret the results. The preoperative cognitive status will serve as a baseline for postoperative cognitive status as well as alpha power in EEG recordings. The musical background will be assessed as a possible explanation for the success of the live or recorded music interventions. Other complications (classified according to Clavien-Dindo [33], nursing-sensitive outcomes such as dehydration and falling [34]) will be explored for changes in the music groups compared to care-as-usual to derive hypotheses for future studies.

### Statistical Analysis

The primary outcomes of this study are VAS subject pain ratings. The *F* test is described immediately below. Statistics will be conducted with SPSS (IBM Corp) or equivalent software [36].

### Primary Study Parameters

Pain ratings will be collected 4 times a day with a VAS. VAS mean and SD will be reported, and box plots, histograms as well as *P* values for Kolmogorov-Smirnov tests will be evaluated for normality of distribution. A univariate, one-way repeated measures (RM) ANOVA will be conducted with factors group (live music intervention, recorded music active control, and standard care control) by time points (1, 2, 3, 4). The α significance threshold will be .05. The result of interest is the significance of the training×time point interaction, that is, whether the course of the data across time points was altered by the training type. Data are planned with 4 time points; in case of up to 2 missing time points, data will be interpolated [37]. Cases with more than 2 missing time points will be excluded from the analysis. Alternatively, if the residuals of the ANOVA are not normally distributed, a similar Kruskal-Wallis one-way ANOVA will be conducted.

### Secondary Study Parameters

For metrics that are collected on the same schedule as the VAS (EEG alpha power, delta/alpha power ratio, entropy metrics, HR, respiration rate, and blood pressure), the values per participant will be entered into the same *F* test described for the VAS ratings (as well as correlated with other baseline parameters as described in the “Other Parameters” section). For blood marker metrics that have 2 data collection points, a similar RM ANOVA will be conducted, but with 2 as opposed to 4 measures, and a significant interaction between treatment and time point would indicate whether treatment significantly impacted the amount of the neuroinflammatory blood marker. Categorical incidence metrics (delirium and POCD) will be evaluated with a one-way ANOVA to determine whether treatment impacted these neuroinflammatory-related complications. Delirium and signs of delirium will be perceived using the DOSS. The DOSS is a screening instrument for delirium, consisting of 13 items, and will be filled by a registered nurse during hospital admission until discharge. Nurses have frequent contact with the patients and are in a good position to observe behavior changes 24 hours a day when patients are admitted. The DOSS is already part of a routine in the surgical wards and all registered nurses are already trained. Likewise, scalar pain medication dosage will be averaged over 24-hour periods per patient and entered into a chi-square test to determine whether the dosage was significantly different across the 3 treatment groups. As this is exploratory and serves to inform future hypothesis-directed testing, we will not correct for multiple comparisons.

### Other Study Parameters

Posthoc analyses will be conducted to inform recommendations for participant grouping in future studies. Continuous baseline parameters (eg, age; see “Other Parameters” section for a full list) will be treated as covariates in an ANOVA in order to see whether the addition of these elements improves the model for each outcome. Similarly, categorical baseline parameters (eg, type of surgery) will be coded and entered as an additional factor (two-way RM ANOVA: treatment×baseline×time point) to see whether the additional factor can explain more variance than in the primary analysis model.

#### Power

The sample size was calculated using G*Power 3.1 [38], an industry-standard software. Below, we explain the parameters for an RM between-subjects univariate ANOVA as well as the final sample size. The calculation was based on the desired ability to detect a within-between interaction (intervention group×time point), with recommended internal settings from [39], which would effectively indicate whether the live music intervention altered the slope of outcome measurements compared to the recorded music or do-nothing control groups.

The effect size (partial η^2^=0.051, converted to f=0.2318206) was determined from a retrospective RM ANOVA with published data [1].

- Alpha: As we have a single quantitative outcome measure, alpha may remain at the conventional .05 significance threshold.
- Power: Following recent recommendations to set power above the typical 0.8 to 0.9 and higher [40], the threshold was set at 0.9.
- Number of groups or measurements: The design calls for 3 groups, with 4 RM (see “Study Design” section; before treatment, after treatment, 3-hour, and 6-hour follow-up) for each participant.
- Nonsphericity correction: As mentioned earlier, retrospective analyses were conducted with the data published in [1]. The ANOVA conducted with that data passed the assumption of sphericity (*P*=.07); therefore, no correction (1) was entered in the G*Power calculation.
- Sample size: The sample size (n=327, Table 3) outputted from G*Power represents the total number of participants in a 3-group design required to achieve 0.9 power with an effect size of ~0.232-109 per group. Padding for a 10% dropout rate raises the per group n to 120, for a total N of 360.

**Table 3:** G*Power sample size calculation.

Parameters			Value
**Input parameters**			
	Effect size *f*(*V*)	0.2318206	
	α err prob	.05	
	Power (1−β err prob)	0.9	
	Number of groups	3	
	Number of measurements	4	
	Nonsphericity correction ε	1	
**Output** **parameters**			
	Noncentrality parameter λ	17.5732385	
	Critical *F*	2.1078920	
	Numerator df	6.0000000	
	Denominator df	972	
	Total sample size	327 (109 per group)	
	Actual power	0.9007901	

## Results

The medical ethics committee of the UMCG approved the study in April 2022. This study began recruiting its participants in June 2022 and upon submission had recruited and collected data from 5 participants. Preliminary data analysis will commence once enough data have been collected to reach 0.8 power. Data collection is expected to be finished in 2026. The study results will be available within 1 year of completed data collection and shared in local, national, and international meetings within the medical, scientific, hospital administrative, and medical professional communities as well as the general public.

## Discussion

### Principal Findings

Previous studies have shown that postoperative pain management can be supplemented with music intervention. Yet, little is known about the benefits of live versus recorded music, or what physiological mechanisms may be mitigating pain perception as a response to listening to music. This NCT will compare the MiMiC live music intervention with listening to recorded music and care-as-usual. The primary objective is to see whether pain VAS ratings differ across the 3 conditions, and the secondary objective is to see whether postoperative neuroinflammation is reduced as a response to listening to music.

The strength of this study is the inclusion of preoperative assessments to establish some baseline characteristics of patients. First, the preoperative musical background questionnaire (selected items from [30]) assesses the estimated weekly hours of music listening as well as listening preferences for musical genre and whether or how the participant is musically trained. This provides us the opportunity to analyze how the extent of musical exposure may affect the primary outcome (pain VAS) of the intervention; depending on the strength of these findings, we may be able to even help inform future music interventions according to preoperative musical habits. Second, the preoperative cognitive assessment (6CIT) [28] serves as an important baseline for several secondary outcomes that may help to account for variability among the 3 intervention arms that otherwise might be attributed to the treatment. Specifically, the 6CIT will inform the interpretation of the postoperative cognitive status, and it may predict alpha power in EEG recording [22] that in turn may be linked to delirium [23,24]. If this proves to be the case, the preoperative cognitive status might be investigated in the future as a method to inform more at-risk patients who could especially benefit from supplementary nonpharmacological interventions [41] such as live or recorded music [42].

### Limitations

While postoperative pain is common to most patients undergoing surgical intervention in hospital, other characteristics surrounding the surgery (eg, type of surgery, duration of surgery, and underlying medical need for surgery) are widely varied across patients. Thus, a limitation of the study is that we will have a heterogeneous patient group. Considering that reduced pain VAS among patients experiencing MiMiC live music intervention compared to care-as-usual was previously a robust finding in a heterogeneous patient group [1], we do not consider our primary outcome to be affected by this heterogeneity. However, secondary outcomes related to finding neurophysiological markers of inflammation, and whether they are influenced by a music intervention, might be greatly affected by this heterogeneity. Moreover, the short duration of the intervention may further limit proper interpretation of inflammatory biomarkers, since previous studies have found neuroinflammatory biomarkers readings to be informative to overall interpretation 5 to 14 days after a medical event [43,44]. We may therefore form posthoc groups with similar surgery types when evaluating secondary outcomes, which could inform future hypotheses for further, longer term clinical research on music interventions and postsurgical neuroinflammation.

### Conclusions

Upon completing this study, we will contribute a systematic comparison of the effects of live versus recorded music on postoperative pain. This study can ultimately inform policy makers as to the benefits to patient pain outcomes when incorporating live versus recorded music in postoperative recovery settings.

## Abbreviations

6CIT: 6-item cognitive assessment
CRP: C-reactive protein
DMBQ: Dutch Musical Background Questionnaire
DOSS: delirium observation screening scale
EEG: electroencephalogram
HR: heart rate
IL6: interleukin-6
MiMiC: Meaningful Music in Healthcare
NCT: nonrandomized controlled trial
NGAL: neutrophil gelatinase–associated lipocalin
POCD: postoperative cognitive decline
RM: repeated measures
TPSO: translocator protein
UMCG: University Medical Center Groningen
VAS: visual analog scale

## Data Availability

When results from this trial are available, data sets will be deposited in a publicly available repository.
